# Responses of Plant Proteins to Heavy Metal Stress—A Review

**DOI:** 10.3389/fpls.2017.01492

**Published:** 2017-09-05

**Authors:** Md. Kamrul Hasan, Yuan Cheng, Mukesh K. Kanwar, Xian-Yao Chu, Golam J. Ahammed, Zhen-Yu Qi

**Affiliations:** ^1^Department of Horticulture, Zhejiang University Hangzhou, China; ^2^Department of Agricultural Chemistry, Sylhet Agricultural University Sylhet, Bangladesh; ^3^State Key Laboratory Breeding Base for Zhejiang Sustainable Pest and Disease Control, Institute of Vegetables, Zhejiang Academy of Agricultural Sciences Hangzhou, China; ^4^Zhejiang Institute of Geological Survey, Geological Research Center for Agricultural Applications, China Geological Survey Beijing, China; ^5^Agricultural Experiment Station, Zhejiang University Hangzhou, China

**Keywords:** heavy metals, phytochelatins, metallothioneins, protein quality control system, ubiquition proteasome system, autophagy

## Abstract

Plants respond to environmental pollutants such as heavy metal(s) by triggering the expression of genes that encode proteins involved in stress response. Toxic metal ions profoundly affect the cellular protein homeostasis by interfering with the folding process and aggregation of nascent or non-native proteins leading to decreased cell viability. However, plants possess a range of ubiquitous cellular surveillance systems that enable them to efficiently detoxify heavy metals toward enhanced tolerance to metal stress. As proteins constitute the major workhorses of living cells, the chelation of metal ions in cytosol with phytochelatins and metallothioneins followed by compartmentalization of metals in the vacuoles as well as the repair of stress-damaged proteins or removal and degradation of proteins that fail to achieve their native conformations are critical for plant tolerance to heavy metal stress. In this review, we provide a broad overview of recent advances in cellular protein research with regards to heavy metal tolerance in plants. We also discuss how plants maintain functional and healthy proteomes for survival under such capricious surroundings.

## Introduction

Proteins are functionally versatile macromolecules that constitute the major workhorses of living cells. They function in cellular signaling, regulation, catalysis, intra and inter cellular movement of nutrients and other molecules, membrane fusion, structural support and protection (Amm et al., 2014). The function of a protein is basically determined by its structure, which is acquired following ribosomal synthesis of its amino acid chain. In addition, the conformation of a protein largely depends on the physical and chemical conditions of the protein environment as affected by extreme temperatures, reactive molecules, heavy metal (HM) ions and other stresses that not only disrupt the folding process of a newly synthesized protein, but also induce the mis-folding of already existing proteins (Goldberg, 2003; Amm et al., 2014; Zhou et al., 2016).

Over the last several decades, the emission of pollutants into the environment has been increased tremendously due to rapid industrialization, urbanization and excessive usage of agricultural amendments. Being sessile, plants are routinely confronted by a wide array of biotic and/or abiotic stresses including HM stress (Al-Whaibi, 2011). HMs are thought to obstruct the biological functions of a protein by altering the native conformation through binding on it (Hossain and Komatsu, 2013). For example, in yeast, methyl-mercury (MeHg) strongly inhibits L-glutamine: D-fructose-6-phosphate aminotransferase, and overexpression of this enzyme confers tolerance to MeHg (Naganuma et al., 2000). Similarly, cadmium (Cd) can inhibit the activity of thiol transferase leading to oxidative damage, possibly by binding to cysteine residues in its active sites. In *Brassica juncea*, Cd-dependent changes in beta carbonic anhydrase result in the enhancement of photorespiration which may protect photosystem from oxidation (D'Alessandro et al., 2013). The modifications caused by Cd disrupt the stabilizing interactions associated with changes in the tertiary structure and cause loss of promising functions of that protein (Chrestensen et al., 2000). Fallout dysfunction of protein stimulates the danger of protein aggregation.

The biosynthesis of metal binding cysteine rich peptides that function to immobilize, sequester and detoxify the metal ions is thought to be the central for detoxification of HMs (Clemens, 2006; Viehweger, 2014). Nonetheless, under extreme conditions, metal ions profoundly affect cellular protein homeostasis by interfering with their folding process and stimulate aggregation of nascent or non-native proteins, leading to the endoplasmic reticulum (ER) stress and a decreased cell viability. To restrict the aggregation as well as to mend them is-folded proteins, cells initiate different quality control systems that fine-tune protein homeostasis. In the center of the system, a typical set of proteins, called heat shock proteins (HSPs; Amm et al., 2014), function as surveillance mechanisms, which are preferentially expressed under stress to maintain functional and healthy proteomes. In contrast, the damaged proteins that fail to achieve their native conformations are subjected to degradation through the ubiquitinproteasome process (UPS), called as ER-associated degradation (ERAD) or through autophagy to minimize the accumulation of misfolded proteins in cells (Liu and Howell, 2016). Although a significant progress has been made in our understanding of protein quality control systems, information on plant system, especially pertaining to HMs stress still remain scanty. In this review, we aim to provide a better insight into the protein quality control system in plants with regards to heavy metal tolerance. We also discuss how plants try to ensure functional and healthy proteomes under HM stress.

## Heavy metals (HMs) detoxification

**Figure 1:**
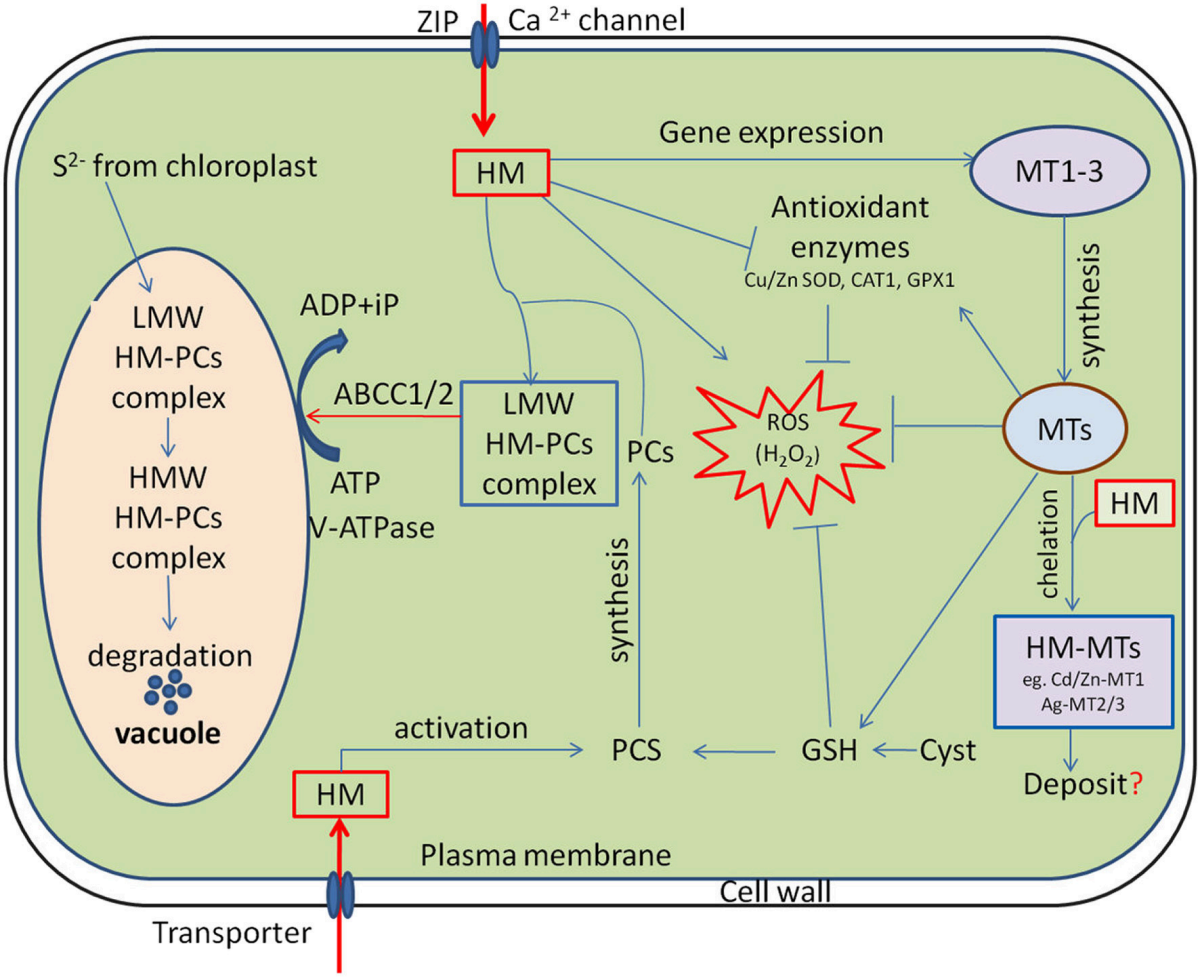
Cellular functions of phytochelatins (PCs) and metallothioneins (MTs) in heavy metal (HM) detoxification. HM activates phytochelatin synthase (PCS) and MTs expression, subsequently the low molecular weight (LMW) HM-PC and HM-MTs complexes are formed in the cytosol. The LMW HM-PCs complexes are consequently transported through tonoplast to vacuole by ATP-binding-cassette and V-ATPase transporter (ABCC1/2). Following compartmentalization, LMW complexes further integrate HM and sulfide (S^2−^, generated by the chloroplasts) to finally form high molecular weight (HMW) HM-PCs complexes. MTs regulates cellular redox homeostasis independently and also by stimulating antioxidant system and stabilizing relatively high cellular GSH concentrations. “→” indicates “Positive regulation” and “-|” represents “Inhibition”, whereas “?” is a “speculation.”

Toxic metal ions at cellular level, evoke oxidative stress by generating reactive oxygen species (ROS; Li et al., 2016a). They promote DNA damage and/or impair DNA repair mechanisms, impede membrane functional integrity, nutrient homeostasis and perturb protein function and activity (Tamás et al., 2014). On the other hand, plant cells have evolved a myriad of adaptive mechanisms to manage excess metal ions and utilize detoxification mechanisms to prevent their participations in unwanted toxic reactions. In the first line of defense, plants utilize strategies that prevent or reduce uptake by restricting metal ions to the apoplast through binding them to the cell wall or to cellular exudates, or by inhibiting long distance transport (Manara, 2012; Hasan et al., 2015). In contrast, when present at elevated concentrations, cells activate a complex network of storage and detoxification strategies, such as chelation of metal ions with phytochelatins and metallothioneins in the cytosol, trafficking, and sequestration into the vacuole by vacuolar transporters (Figure 1; Zhao and Chengcai, 2011).

### Phytochelatins: structure, regulation and function in heavy-metal stress tolerance

In order to reduce or prevent damage caused by HMs; plants synthesize small cysteine-rich oligomers, called Phytochelatins (PCs) at the very beginning of metal stress (Ashraf et al., 2010; Pochodylo and Aristilde, 2017). Notably, PC syntheses play the most crucial role in mediating plant tolerance to HMs (Clemens, 2006; Emamverdian et al., 2015). It has been well documented that the biosynthesis of PCs can be regulated at post-translational level by metal(oid)s in many plant species. However, the over-expression of phytochelatin synthase (PCS) gene in plants does not always result in an enhanced tolerance to HM stress. For instance, over expression of *AtPCS*1 in *Arabidopsis*, paradoxically shows hypersensitivity toward Cd and Zn; although, PCs production is increased by 2.1-folds, when compared with wild type plants (Lee et al., 2003). In reality, excess PCs levels in mutant plants accelerate accumulation of HMs like Cd without improving plant tolerance (Pomponi et al., 2006; Furini, 2012). This phenomenon possibly indicates some additional roles of PCs in plant cells, such as their involvement in essential metal ion homeostasis, antioxidant mechanisms, and sulfur metabolism (Furini, 2012). Therefore, prevention of the free circulation of toxic metal inside the cytosol exhibits a potential mechanism for dealing with HM-induced toxicity (Hasan et al., 2016).

The mechanism of HMs detoxification is not only limited to the chelation, but also involves accumulation and stabilization of HM in the vacuole through formation of high molecular weight (HMW) complexes with PCs (Figure 1; Jabeen et al., 2009; Furini, 2012). Generally, sequestration of metal ions is a strategy adopted by organisms to ameliorate toxicity. The arrested metal ions are transported from cytosol to the vacuole for sequestration *via* transporters. vacuolar sequestration is the vital mechanism to HM homeostasis in plants, which is directly driven by ATP-dependent vacuolar pumps (V-ATPase and V-PPase) and a set of tonoplast transporters (Sharma et al., 2016). RNA-Seq and *de novo* transcriptome analysis showed that different candidate genes that encode heavy metal ATPases (HMAs), ABC transporter, zinc iron permeases (ZIPs) and natural resistance-associated macrophage proteins (NRAMPs) are involved in metal transport and cellular detoxification (Xu et al., 2015; Sharma et al., 2016). A classic example of such protein in Cd uptake in *A. thaliana* is the Fe (II) transporter iron-regulated transporter 1 (IRT1) belonging to the ZIP family (Connolly et al., 2002). Furthermore, NRAMPs members such as NRAMP5 is recognized as an important transporter for Mn acquisition and major pathway of Cd entry into rice roots, which is localized at the distal side of exodermis and endodermis of the plasma membrane of cells (Clemens and Ma, 2016). Interestingly, another transporter HMA2 localized in the plasma membrane of pericycle cells is thought to transport Cd from the apoplast to the symplast to facilitate translocation via the phloem in rice, whereas HMA3 in the tonoplast sequesters Cd into vacuoles by serving as primary pump (Clemens and Ma, 2016; Sharma et al., 2016). The HM transporter 1 (*HMT1*) was first identified in 1995 in the yeast *S. pombe*, as a vacuolar PC transporter required for Cd tolerance (Mendoza-Cózatl et al., 2011). The *HMT1* gene encodes ATP-binding cassette (ABC) membrane transport proteins; therefore, both *HMT1* and ATP are required for the translocation of LMW PC-Cd complexes into the vacuole (Figure 1; Cobbett and Goldsbrough, 2002). In progression, two ABCC subfamily members of ABC transporters, *ABCC1* and *ABCC2* were also identified as additional vacuolar metal-PC complex transporter in *S. pombe* and *Arabidopsis* (Mendoza-Cózatl et al., 2010; Park et al., 2012). Using double mutants, Song et al. (2014) demonstrated that vacuolar sequestration by *ABCC*1 and *ABCC2* is necessary for complete detoxification of Arsenic (As) and Cd in *Arabidopsis*. Interestingly, they also reported that the addition of necessary metal ions, such as zinc (Zn), copper (Cu), manganese (Mn) and iron(Fe) to the transport assay further enhances PC_2_ transport efficiency in barley vacuoles, suggesting that PCs might contribute to both the homeostasis of essential metals and detoxification of non-essential toxic metal(loid)s in plants (Song et al., 2014). Although the mechanism how the transporters regulate the sequestration of metal-PCs conjugates to vacuoles is not clear. Very recent, Zhang et al. (2017), for the first time provided evidence that phosphorylation-mediated regulation of *ABCC*1 activity is required for vacuolar sequestration of As. They found that Ser^846^ phosphorylation is required for the As resistance function of *ABCC*1 in *Arabidopsis*.

### Metallothioneins (MTs): structure, regulation and functions in HM tolerance

Alike PCs, MTs are also naturally-occurring intracellular cysteine-rich major metal-binding proteins, which are used by cells to immobilize, sequester, and detoxify metal ions (Capdevila and Atrian, 2011). Although plant MTs have been discovered over last three decades, the precise physiological functions of MTs have not yet been fully elucidated (Liu et al., 2015a). The proposed roles of MTs include (a) participation in maintaining the homeostasis of essential transition metal ions, (b) sequestration of toxic HMs, and (c) protection against intracellular oxidative damage induced by stress (Hossain et al., 2012a).

Transition metals such as Cu, Fe, Mn and Zn are essential for all organisms because they play critical roles in a variety of physiological processes. For example, Cu is required for photosynthesis, respiration, ethylene perception, ROS metabolism and cell walls in plants (Burkhead et al., 2009; Peñarrubia et al., 2010). A number of studies suggested the involvement of plant MTs in the participation of metal ion homeostasis, especially for Cu, during both vegetative growth and senescence. For example, Benatti et al. (2014) demonstrated that the MTs deficient mutants accumulate 45% and 30% less Cu in shoot and root, compared to the WT, while there are no obvious differences in the life cycle between WT and quad-MT mutant plants under various growth conditions. Again, at early vegetative stage, there is no significant difference in Cu uptake in leaves of 4-week-old WT and MT-deficient mutants. However, the concentration of Cu remains twice in leaves of 12-week-old MT-deficient plants compared to leaves of WT. In contrast, the Cu concentration in seeds of MT-deficient plants was less than half compared to the seeds of WT (Benatti et al., 2014). All these results suggest that MTs are not required to complete life cycle, but are important for essential ions homeostasis and distribution in plants.

In general, sequestration of intracellular HMs in eukaryotes also involves binding of HMs with cytosolic cysteine-rich MTs peptides as well as compartmentalization (Sácký et al., 2014). The combination of low kinetic stability and high thermodynamic is the main features of metal-MT complexes, which bind the metals very firmly, while a part of the metal ions is easily exchanged for other proteins (Maret, 2000). Transgenic plants overexpressing MTs genes have been scored for enhanced metal tolerance and they demonstrate modified metal accumulation or distribution strategies (Gu et al., 2015; Liu et al., 2015a; Tomas et al., 2015). In plants, vacuole is considered as the final destination of detoxification of HMs. Although the chelation of metal ions by MTs is well documented, a little is known about the mechanisms of transport of metals-MT complex from the cytoplasm to the vacuole (Yang et al., 2011). Surprisingly, in *ThMT3* (*hispida* metallothionein-like ThMT3) transgenic material, the expression of four genes (*GLR1, GTT2, GSH1*, and *YCF1*) which aid transport of HMs from the cytoplasm to the vacuole is not induced by Cd, Zn, or Cu stress (Yang et al., 2011). These results advocate that metals are not transported into vacuoles, and that*ThMT3* may only regulate HMs accumulation in the cytoplasm.

The biologic functions of MTs have been a perplexing topic ever since their discovery. Many studies have suggested that in addition to the chelation or metal ion homeostasis, MTs play an important role in cellular redox homeostasis under diverse stress conditions (Kang, 2006). Abiotic stresses like HMs induce excessive accumulation of ROS in plants, and cause damages to the cellular macromolecules such as proteins, leading to metabolic and physiological disorders in cells or even cell death (Hasan et al., 2016). Interestingly, MTs have been proposed as an alternative tool by which plants protect themselves from stress-induced oxidative damage (Figure 1; Hassinen et al., 2011; Ansarypour and Shahpiri, 2017). Although many reports have indicated the roles of MTs in abiotic stress tolerance as ROS scavengers, the mechanisms through which MTs mediate ROS homeostasis remain unclear (Hassinen et al., 2011). It has been proposed that during ROS scavenging, metals are released from MTs and ROS moiety is bounded to the Cys residues of the same. A number of studies have also advocated that the released metals might be involved in the initiation of signaling cascade required for ROS scavenging (Hassinen et al., 2011). For example, normal cellular functioning requires Zn mobilization and its transfer from one location to another or from one Zn-binding site to another. The released Zn from MT mobilized by an oxidative reaction may either constitute a general pathway by which Zn is distributed in the cell or be restricted to conditions of oxidative stress, where Zn is essential for antioxidant defense systems (Kang, 2006), suggesting an important role of MTs in ROS homeostasis and protection of cellular macromolecules from stress-induced ROS. Additionally, the different classes of MTs have distinct tissue-specific expression patterns in plants. As example, GUS reporter constructs explored that *MT1a* and *MT2b* are expressed in the phloem, whereas *MT2a* and *MT3* in the mesophyll cells of young leaves and in root tips (Hassinen et al., 2011). Likewise, Liu et al. (2015a) also demonstrated that *OsMT2c* gene encoding for type 2 MT expressed in the roots, leaf sheathes, and leaves of rice, whereas its weak expression was observed in seeds. Considering their diversified role and tissue specific expression, recently Irvine et al. (2017) showed an excellent effort to develop a low-cost MT-biosensor that can dramatically increase the signal associated with a metal of interest. Such a simple sensor technology could be potentially used in environmental monitoring specially in the areas with the metal contamination problems.

## Repairing of damaged proteins

Proteins are the primary targets of HMs. They either form a complex with functional side chain groups of proteins or displace essential ions from metallo proteins, leading to impairment of physiological functions (Tamás et al., 2014). In addition, HMs interfere with the native confirmations of proteins by inhibiting folding process of nascent or non-native proteins that manifest both in a quantitative deficiency of the affected proteins and in the formation of proteotoxic aggregates (Bierkens, 2000; Tamás et al., 2014). Interestingly, plants inherently respond to stress by triggering the activation of the genes involved in cell survival and/or death in contaminated environments (Hossain et al., 2013). As a part of this plant response ubiquitously involves a set of genes, commonly termed as stress genes, are induced to synthesize a group of proteins called HSPs (Gupta et al., 2010). In stress conditions, the induced synthesis of HSPs plays a significant role in maintaining the cellular homeostasis by assisting accurate folding of nascent and stress accumulated misfolded proteins, preventing protein aggregation or by promoting selective degradation of misfolded or denatured proteins (Hüttner et al., 2012; Park and Seo, 2015).

**Figure 2:**
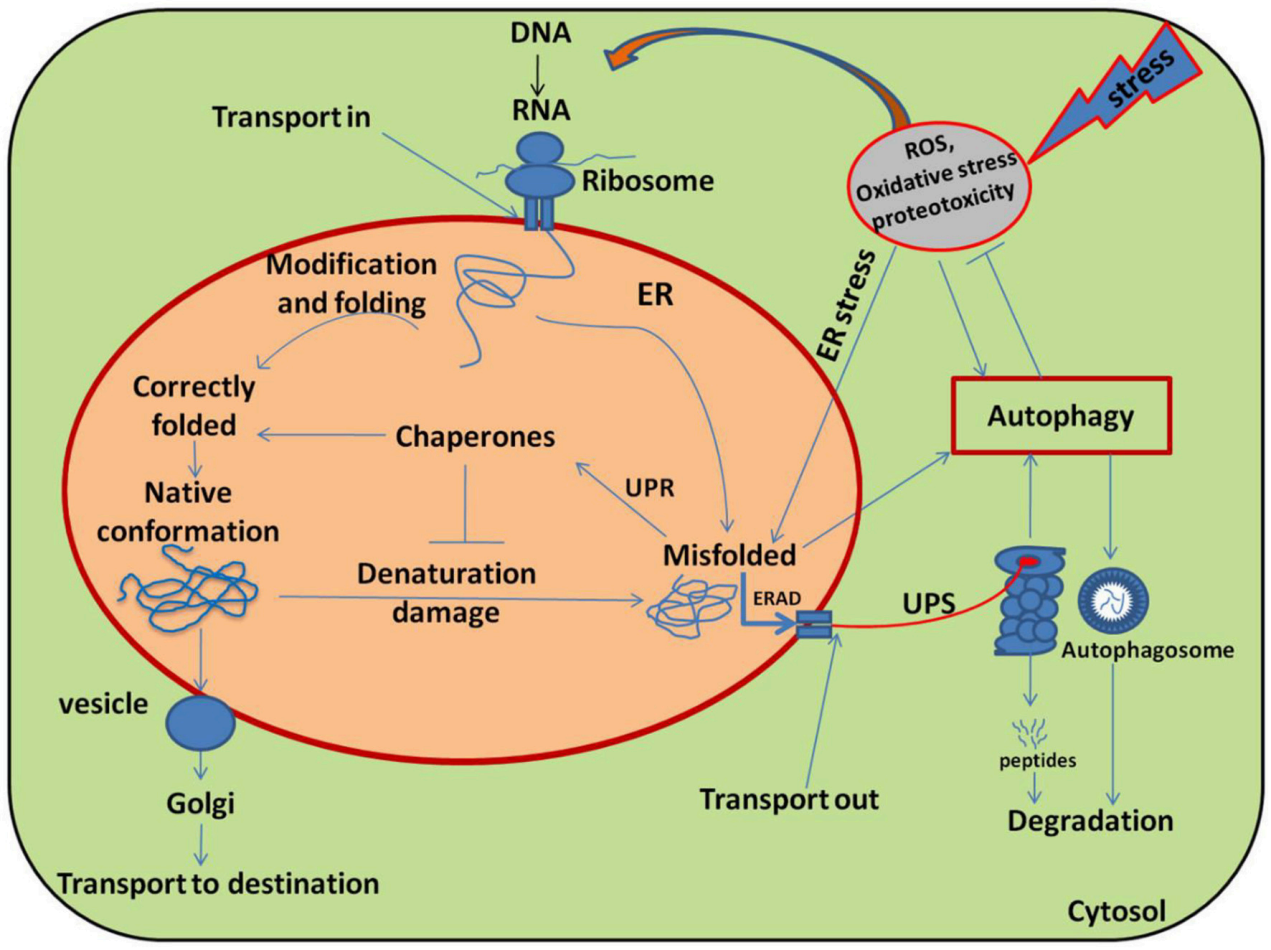
Schematic diagram illustrating the main pathways and regulation of protein folding and modification in the endoplasmic reticulum (ER). Many newly synthesized proteins are translocated into the ER, where proteins folded into their native three dimensional structures with the help of chaperones. The correctly folded proteins are then transported to the Golgi complex, followed by delivery to the destination where they eventually function. While exposure of plants to stress causes oxidative stress by generating over ride of ROS and stimulating the misfolding of proteins. The incorrectly folded proteins are then detected by quality control system, which stimulates another pathway called unfolded protein response (UPR). The terminally misfolded proteins are then eliminated through the endoplasmic reticulum associated degradation (ERAD) pathway, where they initially ubiquitinated and then degraded in the cytoplasm by proteasome system (UPS) or subjected to autophagy. Adopted from Dobson (2003) with modifications.

In fact, HSPs functions as molecular chaperones; proteins which are involved in “house-keeping” inside the cell (Sørensen et al., 2003). Several classes of HSP have been identified in plants (Table 1) and the HSP proteins having molecular weights ranging from 10 to 200 KD are characterized as chaperones which participate in the induction of the signal in stress conditions (Schöffl et al., 1999). For example, in endoplasmic reticulum (ER) all the nascent polypeptides, firstly, are stabilized by chaperones (HSP40 and HSP70-like proteins) such as ERdj3 and binding protein (BiP) before they are properly modified and folded, which prevents aggregation and helps their proper folding (Figure 2; Howell, 2013).

**Table 1:** Five major classes of heat shock proteins (HSPs) that are induced in response to heavy metal stress in plants.

**HSP classes**	**Members**	**Plant species**	**Metals**	**References**
HSPs70	HSP70	*Populus trichocarpa, Lycopersicon peruvianum L., Glycine max, Arabidopsis thaliana, Populus tremula × P. alba, Populus tremula, Populus nigra*	Cd	Lomaglio et al., 2015 Neumann et al., 1994 Hossain et al., 2012b Sarry et al., 2006 Durand et al., 2010 Kieffer et al., 2008 Lomaglio et al., 2015
		*Elodea canadensis Michx*	Cd Pb	Sergio et al., 2007
		*Conocephalum conicum*	Cd, Pb, Cu	Basile et al., 2013
		*Lemna minor*	Cu Cd, Pb, Cr, Zn	Basile et al., 2015
		*Oriza sativa*	As	Chakrabarty et al., 2009; Rai et al., 2015
		*Suaeda salsa*	Hg	Liu et al., 2013
	HSP 70,BiP	*Populus alba L*	Cu, Zn	Lingua et al., 2012
	HSP70	*Oriza sativa, Suaeda salsa*	Ag	Chen et al., 2012; Liu et al., 2013
	HSP68	*Solanum lycopersicum*	Cd	Rodríguez-Celma et al., 2010
	BiP	*Oriza sativa*	Cu,	Ahsan et al., 2007a
	BiP	*Oriza sativa*	Cd	Ahsan et al., 2007b
	HSP 70	*Enteromorpha intestinalis*	Cu	Lewis et al., 2001
	HSPs 70A	*Chlamydomonas acidophila*	Fe, Zn	Spijkerman et al., 2007
	HSP70	*Oryza sativa L*.	Cr	Dubey et al., 2010
	HSC70	*Phytolacca Americana*	Cd	Zhao et al., 2011
	HSC70-2	*Raphanus sativus*	Cr	Xie et al., 2015
HSPs 60	cpn60^2^	*Oriza sativa*	Hg	Chen et al., 2012
	HSP60, Cpn60-B	*Solanum lycopersicum*	Cd	Rodríguez-Celma et al., 2010
	HSP60	*Arabidopsis thaliana*	Cd	Sarry et al., 2006
	HSP60	*Chlamydomonas acidophila*	Fe, Zn	Spijkerman et al., 2007
HSPs 90	HSP90-1	*Lemna gibba*	Cu	Akhtar et al., 2005
	HSP90-1	*Arabidopsis thaliana*	As	Haralampidis et al., 2002
	HSP81-2	*Oryza sativa L*.	Cu	Song et al., 2013
	HSP82	*S. cerevisiae*	As, Cu, Cd	Sanchez et al., 1992
	HSP81.2, 81.3, 81.4, 88.1 & 89.1	*Arabidopsis thaliana*	Cu, Cd, Pb, As	Milioni and Hatzopoulos, 1997
	HSP90-1	*Solanum lycopersicum*	Cr, As	Goupil et al., 2009
	HSP82	*Oryza sativa*	As	Chakrabarty et al., 2009
	HSP81-1	*Oryza sativa*	Cd	Oono et al., 2016
HSPs 100	HSP104	*S. cerevisiae*	As, Cu, Cd	Sanchez et al., 1992
	HSP101	*Oryza sativa L.*,	As	Agarwal et al., 2003
	ClpB-C	*Oryza sativa L*.	Cans, Cu, Co	Singh et al., 2012
	ClpB-C	*Arabidopsis thaliana*	As	Mishra and Grover, 2014
sHSPs	HSP17	*Lycopersicon peruvianum L*,	Cd	Neumann et al., 1994
	HSP17	*Populus alba L*	Cu, Zn	Lingua et al., 2012
	HSP21	*Arabidopsis thaliana*	Cd	Zhao et al., 2009
	HSP20,HSP23p	*Kandelia candel*	Cd	Weng et al., 2013
	HSP26.13p	*Chenopodium album*	Ni, Cd, Cu	Haq et al., 2013
	HSP17	*Armeria maritime*	Cu	Neumann et al., 1995
	HSP17	*Silene vulgaris, Lycopersicon peruvianum*	Hg, Cu, Cd,	Wollgiehn and Neumann, 1999
	HSP24	*Capsicum annuum L*	Zn	Zhu et al., 2013
	HSP22	*Chlamydomonas acidophila*	Cu	Spijkerman et al., 2007
	HSP17.4	*Oryza sativa L*.	Fe, Zn	Dubey et al., 2010
	HSP20, HSP21, HSP22	*Raphanus sativus*	Cr	Xie et al., 2015
	HSP23	*Glycine max*	Cr	Zhen et al., 2007
	HSP23.9	*Oryza sativa*	Al	Chakrabarty et al., 2009
	HSP17.4, HSP17.5	*Tamarix hispida*	As	Gao et al., 2012
	HSP18.3	*Oryza sativa*	Cu, Cd, Zn	Rai et al., 2015

### Role of HSPs in plant tolerance to HM stress

HM stress often causes disturbance to the cellular homeostasis by inactivating essential enzymes and by suppressing proteins functioning (Hossain et al., 2012b). Hence, the induction of HSPs proteins is thought-out as a critical protective, eco-physiologically adaptive and genetically conserved response of organisms to the environmental anxiety. Thus, they accomplish a key function in the hostility of stress by re-establishing normal protein conformation and cellular homeostasis (Rhee et al., 2009). Among the major categories of HSPs, HSP70 family members have extensively been studied. Functional characterization of HSP70 revealed that HSP70 is accumulated in response to environmental stressors in a wide range of plant species (Gupta et al., 2010). The specific members of this family are localized into the cytosol, mitochondria and endoplasmic reticulum(ER) and are constitutively expressed as well as regulated to maintain cellular homeostasis. An example to cite, the 70-KDa heat shock cognates(HSC70) are constitutively expressed in cells and often assist in the folding of *de novo* synthesized polypeptides and import or translocations of precursor proteins (Wang et al., 2004).

The recent advancements in proteomics research have enabled us to identify the functional genes or proteins involved in the responses of plants to HM stress at molecular levels (Ahsan et al., 2009). Transcript analysis in many plant species showed that HSP70 is highly expressed under a variety of metal stress (Table 1). Although many studies showed that the over-expression of HSP70 genes is positively correlated with the acquisition of tolerance to various stresses, including HMs, but the cellular mechanisms of HSP70 function under stress situation are not completely understood (Wang et al., 2004). HSP70 chaperones, together with their co-chaperones like DnaJ, make a set of prominent cellular machines to prevent accumulation of newly synthesized proteins as aggregates and ensure the proper folding of protein during their transfer to the destination (Al-Whaibi, 2011; Park and Seo, 2015). In transportation of precursor protein, the HSC70 is essential for cell-to-cell transport through interaction with the plasmodesmatal translocation pathway (Aoki et al., 2002). The induction of HSP70 not only limits the proteotoxic symptoms of metalions, but also helps the sequestration and detoxification of these ions by MTs (Haap et al., 2016). While the entire mechanism of HSPs-induced metal detoxification via MT has yet to be explored, only few studies pointed out that HSP60 might participate in protein folding and aggregation of many other proteins that are transported to organelles such as mitochondria and chloroplasts (Al-Whaibi, 2011). With our increasing understanding of the proteome, it is becoming clear that HSP60 is essential for cellular functions both at normal or stress environments, including metal stress (Table 1). Interestingly, proteomics analysis also revealed that the induction HSP60 chaperones prevents the denaturation of proteins even in the presence of metal ions in the cytoplasm (Sarry et al., 2006; Rodríguez-Celma et al., 2010). Similarly, a good number of studies showed the induction of HSP90 family proteins by different metals in many plant species (Table 1) which play a major role in protein folding and regulating signal-transduction networks, cell-cycle control, protein degradation and protein trafficking (Pratt and Toft, 2003; Al-Whaibi, 2011). Interestingly, they have also been found in association with several other intercellular proteins, including calmodulin, actin, tubulin and some other receptors and signaling kinases (Wang et al., 2004; Gupta et al., 2010; Park and Seo, 2015). The multiple sites of localization and high accumulation in combination with other intercellular proteins lead to the suggestion that these polypeptides perform a general mode of cellular activities (Prasad et al., 2010). This family of proteins might provide genetic buffering and contribute to the evolutionary adaptation of plant both in normal and stressful conditions (Wang et al., 2004). By contrast, there is no substantial evidence implicating HSP100/Clp proteins in HMs tolerance in plants (Agarwal et al., 2003). Recently, few studies reported that many members of this family are induced in response to metal treatments (Table 1), and they accomplish house keeping functions necessary for cellular homeostasis (Lee et al., 2006).

Most of the members of sHSPs are strongly inducible and some are also constitutively expressed under environmental stress conditions. One of the featured functions of this family of protein is the degradation of the proteins not suitable for folding (Gupta et al., 2010). Similar to other HSPs, sHSPs also function as molecular chaperones, however, the important characteristic that distinguishes sHsps from other chaperone classes, such as DnaK or ClpB/DnaK is that their activity is independent of ATP (Sun et al., 2002; Mogk and Bukau, 2017). sHSPs maintain denatured proteins in a folding-competent state and allow subsequent ATP-dependent disaggregation through the HSP70/90 chaperone system, thereby facilitating their subsequent refolding (Wang et al., 2004). Recently, it has been shown that BAG3 protein acts as a modulator, brings the chaperone families together into a complex and coordinates the potentiality of Hsp22 and Hsp70 to refold the denatured proteins (Rauch et al., 2017). Additionally, this family of HSPs is also involved in signaling similar to their counterparts, where they become phosphorylated by stress-kinases, and increase the amount of reduced glutathione in the cytoplasm (Arrigo, 1998). A number of studies suggest a strong correlation between sHSP accumulation and plant tolerance, particularly to HMs stress (Table 1; Wang et al., 2004). In *Arabidopsis*, 13 different sHsps have been identified in distinct cellular compartments, which probably function as mediators of molecular adaptation to stress conditions and are unique to plants. The differential regulation of chloroplastic i.e., Cp-sHSPs or HSP26.13p in *C. album* plays a dual role in protecting the plant from heat and metals like Ni, Cu, and Cd stress (Haq et al., 2013).

Notably, most of HSPs are activated early in the cascade of cellular events following toxic exposure even below the lethal dose and thus, considered as strong biomarkers for environmental pollution (Bierkens et al., 1998). The excessive accumulation of ROS at cellular level is a common consequence of abiotic stress such as HMs. Interestingly, the high HSP levels protect plants against abiotic stresses not only by preventing irreversible protein aggregation, but also by promoting cellular redox homeostasis through stimulating antioxidant systems (Mu et al., 2013). Recently, Cai et al. (2017) revealed that HSPs-induced metal tolerance in plants has a strong correlation with melatonin (N-acetyl-5-methoxy tryptamine) biosynthesis, which is regulated by heat-shock factor A1a (HsfA1a). Therefore, all the above presented results suggest that the inducible HSPs are important and beneficial for fitness in normal, as well as under capricious environment. As a part of protein quality control system, HSPs play a major role in maintaining the functionality of cellular machinery under environmental stress. Therefore, research on the functional and structural aspects, cross-talk with other chaperones and relationship between different HSP expressions along with the physiological stress response should be expanded to better understand their functions.

### Heavy metals-induced ER stress

In cells, both recently synthesized and preexisting polypeptides are at constant risk of misfolding and agreegation. It has been estimated that one-third of recently synthesized proteins are misfolded under ambient conditions (Schubert et al., 2000). In addition, cells continuously face environmental challenges such as mutations, heat, active oxygen radicals and HM ions, which not only disrupt protein folding but also cause the misfolding of already folded protein, (Amm et al., 2014). The disruption of proper functioning of the ER is particularly relevant under stress conditions, whereas the demand for secreted proteins exceeds its working capacity and disrupts normal functioning of ER, termed as ER stress (Schröder and Kaufman, 2005). A number of studies have shown that HMs and metalloids inhibit refolding of chemically denatured proteins *in vitro*, obstruct protein folding *in vivo* and stimulate aggregation of nascent proteins in living cells (Sharma et al., 2011; Jacobson et al., 2012). For example, Cr has been shown to trigger oxidative protein damage and protein aggregation in yeast by enhancing mRNA mistranslation (Sumner et al., 2005; Holland et al., 2007). Likewise, Cd has also been shown to cause the widespread aggregation of nascent protein and ER stress in yeast (Gardarin et al., 2010), whilst the mechanistic details of protein misfolding in the ER and cytoplasm remain to be elucidated (Tamás et al., 2014). But somehow, this could be related to metal-induced structural alteration of ER. Recently, Karmous et al. (2015) demonstrated that Cu treatment in embryonic cells of *Phaseolus vulgaris* results in prevalently swollen cisternae of smooth endoplasmic reticulum and vesicles with electron-dense contents. Although this phenomenon is often not well recognizable, it robustly inhibits cellular homeostasis. While the toxicity of metals, like Cd, As, Pb, Hg, and Cr is unquestionable and their interference with protein folding in living cells is well-documented, the potency of accumulation of misfolded and aggregated proteins appears to be different (Tamás et al., 2014). In yeast cells, the accumulations of aggregated proteins occur in the order As>Cd>Cr upon exposure of equi-toxic concentrations of these metals (Jacobson et al., 2012). The *in vivo* potency of these environmental threats to prompt protein aggregation possibly depends on the efficiency of their cellular uptake/transport and their distinct modes of biological action (Tamás et al., 2014). Under such circumstances, a coordinated adaptive program called the unfolded protein response (UPR) is commonly initiated.

The UPR is a homeostatic response to alleviate ER stress through transcriptional and translational events. The induction of UPR has three aims; initially to restore the normal function of the cell by halting production of secreted and membrane proteins, removal of misfolded proteins through ER-associated degradation (ERAD) systems, and activation of the signaling pathways that lead to increased production of molecular chaperones involved in protein folding. If these cell-sparing activities are not achieved within a certain time span or the disruption is prolonged, then the UPR aims toward programmed cell death (PCD) which is called apoptosis (Deng et al., 2013; Liu and Howell, 2016). The UPR is a sensitive surveillance mechanism that monitors the ER loading capacity. If persistently misfolded proteins exceed the ER loading capacity, cellular communication between the ER and nucleus occurs for ER homeostasis, leading to the transcriptional activation that up-regulates the cellular chaperones (Brandizzi et al., 2014). Recently, it has been reported as two arm process in plants, where proteolytic processing of membrane-associated bZIP TFs and RNA splicing factor inositol-requiring enzyme-1 (IRE1) act as transducers in ER stress or UPR signaling pathway. Structural details of bZIP TFs, bZIP17, bZIP28 and IRE1/ bZIP60 and their underlying principles of mobilization in response to ER stress suggest that the UPR signaling pathway is assisted by these factors in order to protect plants from ER stress(Deng et al., 2013; Sun et al., 2013; Yang et al., 2014). This UPR transcriptional activation enhances the production of molecular chaperones such as binding protein (BiP) and glucose-regulated protein 94 (GRP94), involved in increased ER protein-folding potentiality (Yoshida et al., 1998). The molecular chaperones binding proteins (BiP) are the master regulatory elements of these quality control systems and a classical marker of UPR activation (Malhotra and Kaufman, 2007). For example, Xu et al. (2013) demonstrated that heterologous expression of *AtBiP2* protein in BY-2 act as a negative regulator of Cd-induced ER stress and PCD. Recently, Guan et al. (2015) also showed that ER chaperone binding protein *BiP* acts as a positive regulator in Cd stress tolerance. To explore the mechanism, the author also examined the transcript level of *GSH* gene in *LcBiP*-overexpressed tobacco. Interestingly, the transcript levels of the *GSH* and the ER stress marker gene are not induced, meaning that BiP may enhance the folding capacity of *GSH* in the ER in plants. In contrast, proteins that fail to achieve their native conformation and aggregate in ER are eliminated through the ERAD pathway *via* a series of tightly coupled steps that include substrate recognition, targeting of terminally misfolded proteins, retro-translocations, and ubiquitin-dependent proteasomal destruction (Liu and Howell, 2016). Liu et al. (2015b) discover a plant-specific component of ERAD system in *Arabidopsis*. They uncovered that EBS7 (methanesulfonate-mutagenized brassinosteroid insensitive 1 suppressor 7) interacts with the ER membrane-anchored ubiquitin ligase AtHrd1a, one of the central components of the *Arabidopsis* ERAD machinery, whose mutation destabilizes AtHrd1a to reduce polyubiquitination. Similarly, very recently, Liu et al. (2016) demonstrated that α1, 6-linked mannose is necessary for luminal N-glycoproteins degradation via ERAD system in yeast where Htm1p-Pdi1p complex acts as a folding-sensitive mannosidase for catalyzing the first committed step. Thus, any defects in this sophisticated ERAD machinery system raise death and life issues of cells survival, especially under stressed environments. For instance, Van Hoewyk (2016) demonstrated that *Arabidopsis* HRD1 and SEL1L mutant plants showed decreased tolerance to selenate (Se) stress. As-Se toxicity causes both oxidative stress and protein misfolding due to the substitution of a cysteine to Se-cysteine (Van Hoewyk, 2013), whereas selenium enhances Cd tolerance in tomato plants (Li et al., 2016b). Actually, this is an alternative adaptive mechanism, involved in actively controlled and precise degradation of cellular components, and selective elimination of harmful, unwanted or damaged cells in eukaryotes (Tuzhikov et al., 2011; Brandizzi et al., 2014). Such selective suicide or PCD is paradoxically a crucial event which eventually provides survival benefits for the whole organism under extreme environmental conditions like HMs stress (Yakimova et al., 2007; Adamakis et al., 2011). However, the details of involved mechanisms are still obscure and remain to be further disclosed.

## Degradation of metal-induced denatured proteins

Much of plant physiological processes, growth and development are controlled by the selective degradation of unwanted misfolded or damaged proteins in order to maintain cellular homeostasis. In plants, protein degradation or proteolysis occurs either by ubiquitin proteosome system (UPS) or by autophagosomes induction (Liu and Howell, 2016). The UPS is a fundamental, highly regulated multistep enzymatic cascade that tightly controls the cellular protein homeostasis. It is a very rapid and effective method for a precise degradation of an unwanted protein at a particular time, whereas most of the times a protein is degraded only in response to a specific cellular signal or event (Pines and Lindon, 2005). The recent progress in molecular genetics revealed that like a tip of iceberg, UPS components regulate critical processes in plants (Sadanandom et al., 2012). It has been found that over 6% of *Arabidopsis* protein-coding genes are dedicated to the UPS (Vierstra, 2009). In eukaryotic cells proteins that destined to degrade, firstly, labeled by ubiquitin and then the ubiquitylated protein digested to small peptides by the large proteolytic complex, the 26S proteasome (Goldberg, 2003). Ubiquitylation is an energy dependent key regulatory process, which is executed by three different E1-E2-E3 enzymes conjugation cascade (Maupin-Furlow, 2013). In the first step of this cascade, ubiquitin is covalently conjugated to ubiquitin-activating enzyme (E1) in an ATP-dependent reaction, and then this activated ubiqutin is transferred from E1 to E2 (or Ub-conjugating enzyme) by transesterification. Finally, E3 (ubiquitin ligase) catalyzes the transfer of the ubiquitin from the E2 enzyme to a lysine residue on the target protein. After initial polyubiquitination, this step is repeated to form polyubiquitinated chains on the target protein and designated for degradation by the 26S proteasome (Ruschak et al., 2011; Sadanandom et al., 2012). Upon delivery, the deubiquitinating enzymes remove the polyubiquitin chain before unfolding, import and proteolysis of targeted substrates (Hartmann-Petersen et al., 2003).

On the other hand, autophagy is a biological self-destruction process, by which eukaryotic cells maintain their cellular homeostasis by turning over damaged proteins or organelles into the vacuole during developmental transitions and under stress conditions (Liu and Bassham, 2012; Wen-Xing, 2012). Upon the induction of an autophagic pathway, the cytoplasmic components that designate to degradation, are surrounded by a double membrane structure, called autophagosomes (Figure 2). The autophagosome then delivers its cargo materials to the vacuole, where the outer membrane of the autophagosome initially fuses with the vacuole membrane, after which the cargo materials are degraded by vacuolar hydrolases in the vacuole and recycled (Yang et al., 2016). There are four different types of autophagy detailed out in eukaryotes, including microautophagy, macroautophagy, chaperone-mediated autophagy and organelle-specific autophagy (Liu and Bassham, 2012). Out of these, microautophagy and macroautophagy (hereafter referred as autophagy), have been shown to occur in plants (Han et al., 2011).

### UPS-dependent proteasome activity and metals stress

Extreme environmental conditions such as HMs pollution often adversely affect proteins quality by increasing free radicals that encourage denaturation and damage, thus causing the misfolding of proteins. Cells under stress, need to prevent further damage by initiating defense machinery to repair the damaged proteins or remove them when irreparable. In such circumstances, the UPS plays a crucial role in plant response and adaptation to changing environmental conditions(Stone, 2014). The UPS, functions both in cytoplasm and nucleus, responsible to modulate the levels of regulatory proteins and to remove most abnormal peptides and short-lived cellular regulators which may accumulate following exposure to abiotic stress (Lyzenga and Stone, 2012). The significant role of the UPS in the cellular response to HM stress has been recognized few years ago and is evident to increased expression of polyubiquitin genes (Genschik et al., 1992; Jungmann et al., 1993). It is believed that the expression of polyubiquitin genes under stress conditions is one of the important indications that the UPS is involved in regulation of plant HMs stress tolerance (Sun and Callis, 1997; Chai and Zhang, 1998). The genome-wide transcription analysis of rice plants showed that low concentrations of Cd treatment induces polyubiqutin expression both in root and shoot (Oono et al., 2016). In contrast, elevated metal concentrations induce the disruption of proteasomal activity, resulting in the accumulation of abnormal proteins in the cytosol, which alter cellular protein homeostais and thereby activating apoptosis (Yu et al., 2011). For instance, proteome analysis in different plant species has shown that ubiquitin activity can significantly be reduced by Cd, Co, Cu, Cr, Hg, Ni and Pb at 100 μM but not by Al and Zn, whereas low concentrations can induce 26S proteosome activity (Aina et al., 2007; Pena et al., 2007, 2008). Although Co, Cu, Cr, Hg, Ni, and Pb induce accumulation of ubiquitin conjugated proteins, the abundance of 20S core protein in UPS system is not changed (Pena et al., 2008). In contrast, Karmous et al. (2014) showed that Cu treatment (200 μM) has a strong effect on UPS pathway and can inhibit about 88% of the 20S proteasome activity in the cotyledons of germinating bean seeds. This implies that the effect of HMs on proteolytic system can not be generalized; however, the functional impairment-induced decrease in proteases activities appears to be a common aspect of metal toxicity in plant.

In extreme environments, over-expression of genes involved in UPS cascade, enhances tolerance to multiple stresses without any adverse effects on growth and development in plants (Guo et al., 2008). For example, Lim et al. (2014) showed the E3 or ubiquitin ligase enzyme is an important regulator for the removal of aberrant proteins under metal stress. In addition, the heterogeneous expression of rice E3 ligase enzyme synthesis RING domain *OsHIR1 gene* in *Arabidopsis* has been found to be decreased with As and Cd accumulation in both root and shoot (Dametto et al., 2015). Although the mechanism is not clear, it could probably be regulated by its substrate protein, since the OsHIR1 protein positively interacts with the OsTIP4;1 related to As and Cd uptake. Therefore, the development of strategies to reduce metal uptake and translocation as well as to improve cellular protein homeostasis with the ubiquitin/proteasome 26S system in plants seems to be a promising approach to ensure crop yield and food safety.

### Autophagy in metal stress responses

Autophagy has shown to be involved in the adaptation of plants to a wide range of drastic environmental stresses such as nutrient starvation, oxidative stress, heat stress, drought, salt, and pathogen invasion (Han et al., 2011; Wang et al., 2015; Xu et al., 2016; Yang et al., 2016). However, its pivotal roles in plants, particularly in HMs stress and adaptive responses, have perhaps not received the attention they deserve and thus remains elusive (Chiarelli and Roccheri, 2012; Pérez-Martín et al., 2015). Fascinatingly, in recent years scientists begun to explore, the involvement of autophagy in plant toward metal tolerance and the mechanism of adaptation is the same as in human and yeast. Firstly, Zhang and Chen (2010), afterwards, Zheng et al. (2012) demonstrated that autophagy is induced in metal treated plants. In progression, the expression profile analysis in tobacco seedlings after five different HMs (Cu, Ni, Zn, Cd and Mn) treatments showed that among the 30 *ATG*s genes, 18 *ATGs* genes are upregulated by more than two folds by at least one HM. They also explored that among the 18 *ATGs*, 11 *ATGs* are commonly up-regulated in seedlings by all five metals, and the expression is more sensitive to Zn treatment than others. Recently, Abd-Alla et al. (2016) for the first time demonstrated that Ag-NPs treatments result in the induction of autophagy in root nodules of *Rhizobium leguminosarum* as a mechanism of detoxification and surveillance. Taken together, recent studies explored the involvement of autophagy as a sophisticated regulator of surveillance under HMs stress, nevertheless, the mechanisms, especially how metals regulate autophagy, still remain to be elucidated in the future (Zhou et al., 2015).

Interestingly, the cellular sites of ROS production and signaling are thought to be primary targets of autophagy, which leads to either survival or death of cells (Scherz-Shouval and Elazar, 2007; Minibayeva et al., 2012). It has now been undoubtedly established that ROS can function as specific second messengers in signaling cascade. Environmental toxicants, like HMs are known to be strong inducers of oxidative stress due to excessive accumulation of ROS that alter the cellular homeostasis. In general excess accumulation of ROS causes many types of cellular injuries including damage to proteins, lipids and DNA, whereas some of which can result in apoptotic cell death and autophagy (Farah et al., 2016). Several lines of evidence suggest that metal-induced intracellular ROS production function in the signal transduction pathways, leading to induction of autophagy (Zhang and Chen, 2010; Pérez-Martín et al., 2015; Farah et al., 2016). Although an extensive number of studies showed that oxidative stress stimulates the autophagic induction to relieve the plants from oxidative damage, but the mechanistic information still remain limited. Very recently, Yang et al. (2016) put forward an excellent effort to open-up the mechanism for activation of autophagy. They demonstrated that unfolded proteins accumulation in the ER is a trigger for autophagy under conditions that cause ER stress. They showed that the reduction of unfolded proteins accumulation by PBA or TUDCA addition or BiP over-expression inhibits the autophagy in *Arabidopsis*. Whereas, the introduction of the constitutively unfolded proteins zeolin or CPY* activates the UPR and autophagy via IRE1b (inositol-requiring enzyme 1b) dependent manner. But how the ER stress activating these signaling cascades remains to be further revealed.

In addition to their core function, autophagic induction has also been shown to be involved in the regulation of metal uptake. For instance, Li et al. (2015) showed that induction of autophagy with mono-ubiquitination under Fe excess condition affects the functional activity and stability of exogenous *Malus xiaojinensis* iron-regulated transporter (MxIRT1) in yeast, thereby preventing Fe uptake via this root transporter. They also showed that in Fe led conditions, the transcript levels of ATG8 and ATG8-PE protein significantly increased, which resulted in enhanced MxIRT1 degradation, while the inhibition of autophagic initiation has opposite effects. Therefore, the development of strategies to regulate metal uptake by promoting autophagy under excess metal conditions could have potential implication in increased or even safe food production. However such kind of assumption in plant is still a matter of speculation and thus requires further extensive investigation.

## Concluding remarks and future perspectives

The present review outlines the impact of HM stress on cellular protein homeostasis and illustrates the diverse mechanistic approach that operates inside cells to regulate quality control systems toward functional and healthy proteomes. Proteins are major workhorse of cells and directly involved in plant stress response. HMs can trigger the cellular pathways that are broadly classified as death and survival signals. As surveillance mechanism, the ubiquitous plants response to HM stress is the chelation of toxic ions in the cytosol by cysteine rich peptides such as PCs and MTs, compartmentalization of metals in the vacuole by tonoplast located transporters, and the process that involves repair of stress-damaged proteins. In-depth review of recent research works revealed that MTs are not only required to complete the plants life cycle, but also play significant roles in ionic homeostasis and distribution in plants as well as cleanup of ROS and sequestration of metals as that of PCs (Benatti et al., 2014; Tomas et al., 2016). Whilst in extreme conditions metals profoundly affect cellular protein homeostasis by interfering with the folding process, they also stimulate aggregation of nascent or non-native proteins leading to ER stress and decreased cell viability. However, there is a typical set of proteins, called stress proteins or HSPs proteins, which are preferentially expressed under stress. HSPs restrict aggregation of nascent or non-native proteins but trigger repair of misfolded proteins. In contrast, the damaged proteins which fail to achieve their native conformations, are removed from ER by the activation of ERAD machinery of ERQC system, leading to proteosomal (UPS) or autophagic degradation.

Recent advances in protein research, summarized herein, show that as core degradation process of misfolded or damaged polypeptides, the over-expression of E3 enzyme in UPS pathway and the autophagic induction with mono-ubiquitination prevent metal accumulation in plants (Dametto et al., 2015; Li et al., 2015). However, we are still far away from the complete understanding of the mechanism of subsequent signaling cascades that regulate metal accumulation. The development of strategies to reduce metal uptake and translocation by manipulating cellular protein quality control system in plants seems to be a promising approach that can potentially ensure increased yield as well as food safety.

## Author contributions

MH and GA conceived the idea and designed the outlines of the article. MH, YC, MK and GA wrote the article. GA, XC, and ZQ revised the article.

### Conflict of interest statement

The authors declare that the research was conducted in the absence of any commercial or financial relationships that could be construed as a potential conflict of interest.
